# Everybody's Page

**Published:** 1891-12-26

**Authors:** 


### Everybody's Page.

It is asserted in some Italian and other medical journals that protection has been afforded by heifer vaccine against measles, whooping-cough, and influenza. It is quite clear, however, that our too great experience of the latter complaint, which bids fair to be increased this coming winter, has not yet taught us either a protection or a remedy. The vagaries of the disease are only equalled by the diversities of opinion as to the mode of its origin and the method of its spread.

Those who have tender feet which become sore after much walking, as many people's do on flag pavements, will experience considerable relief, it is stated, by soaking the feet once or twice a week in, say, two quarts of hot water, containing a piece of nitrate of potassium the size of a large olive. This simple remedy might have proved an untold blessing to the troops who are alleged to have broken down so numerously on the march to their manœuvring grounds during the past summer.

"An Earnest but Critical Friend of Hospitals" writes : This season finds many besides myself puzzling to provide suitable Christmas presents. I believe that one's mind and bounty can be better employed, so enclose cheque, value £20, crossed to credit of the Royal South London Ophthalmic Hospital, St. George's Circus, Southwark. Last Saturday I visited the almost completed new building for this hospital. I found it magnificently placed and well planned. I was struck by the exceptional attention to detail, consideration for efficiency with economy in working, and the great mastery of difficulties evident throughout. I do not like to think that for want of funds such admirable structural facilities for contending against blindness may not be fully and promptly utilised. I shall notify those who will consequently miss receiving a Christmas souvenir, and trust they will follow, and not sneer at my departure from the conventional. Please respect my anonymity, and pass this note to the Hospital's Secretary.

A wife in sore affliction wrote recently to her husband's employer to say that she regretted her husband was unable to come to business as he was ill with the blight. It turned out on enquiry, that this mysterious disease, which is usually associated with fruit and other trees, rather than with man, was the influenza. That it is a blight there can be hardly any gainsaying, and the term hits off the nature of the disease uncommonly well.

A decidedly novel form of fire insurance is reported to be in use in a certain town in Quebec. The merchants who inhabit this remarkable place are evidently more fully endowed with faith than with those capacities which, in the nineteenth century, are looked for in a man of business, for there, instead of paying premiums to insurance companies, they are said to pay a joint annual subscription to the priests for a certain number of masses to be said, which will have the effect of making them quite secure against fire. This is an excellent system—for the priests, but it does not seem to be unreasonable that the wholesale dealers who supply these quixotic folk should disapprove somewhat energetically of their blind and unbusinesslike faith.

The resolutions passed at a recent meeting convened by the Church of England Burial, Funeral, and Mourning Reform Association, that is desirable that the Church should endeavour to bring back the nation to a mode of burial which answers the requirements of sanitary laws, and deprecating modern innovations in respect of mourning and other accompaniments of funerals in so far as they ignore simplicity and economy, are far more practical than resolutions at public meetings often are. The great offenders against simplicity and economy are the poor, and it is only by educating them in the right direction that a remedy can be found ; this can be done partly by example, no doubt, but the idea that too much money cannot be spent on a funeral has become a sort of creed with the uneducated, which the most persuasive arguments will find hard to destroy.

